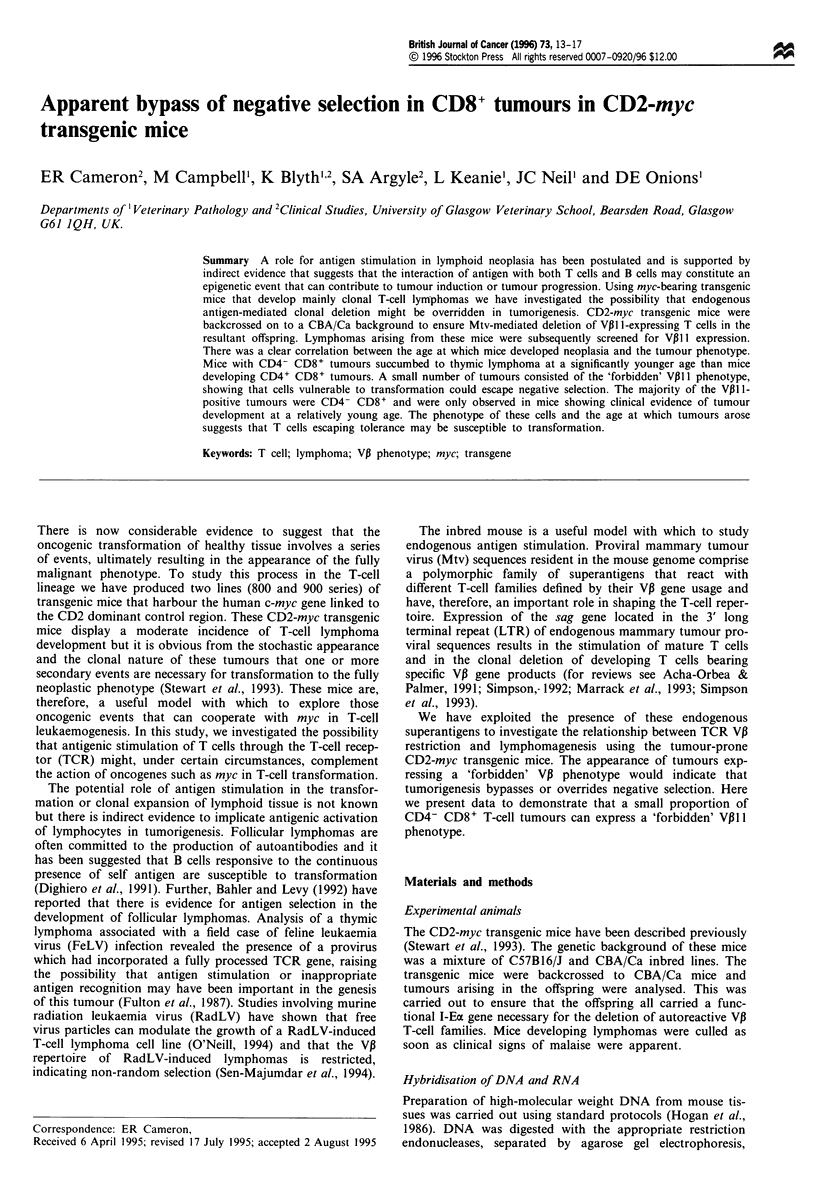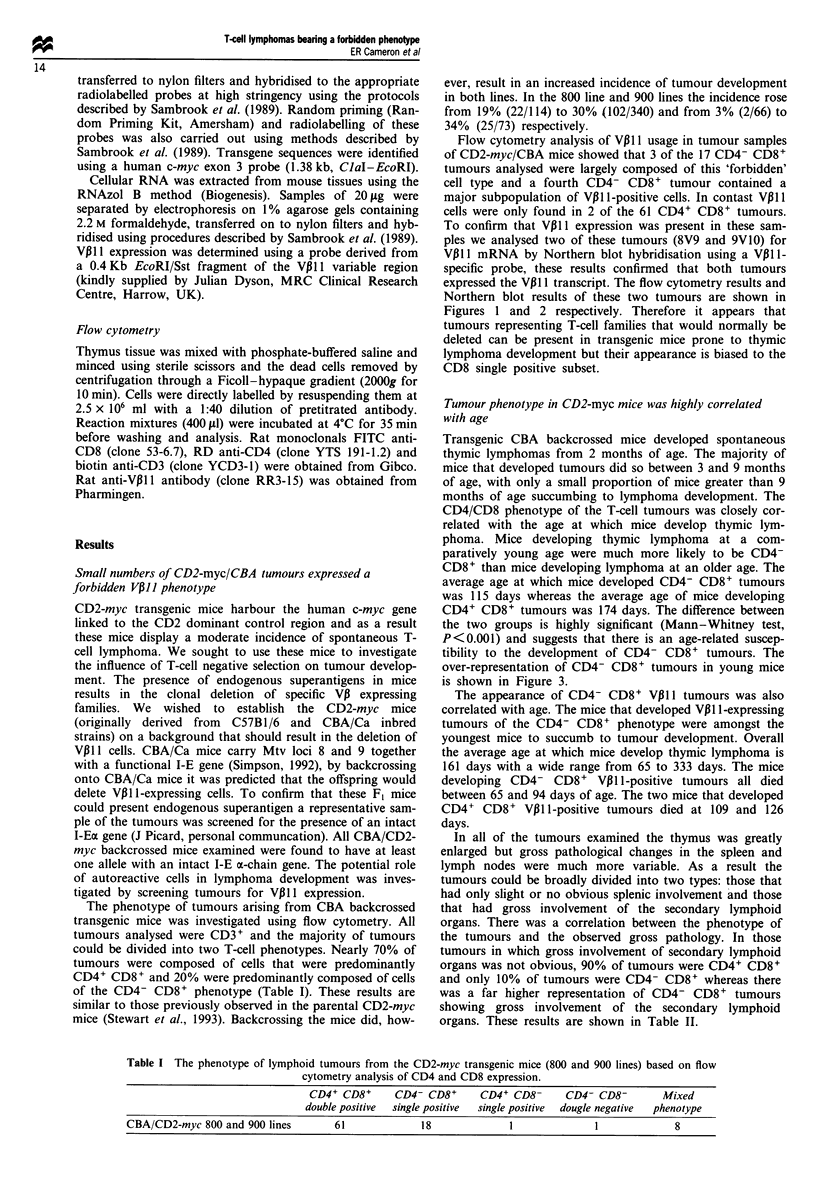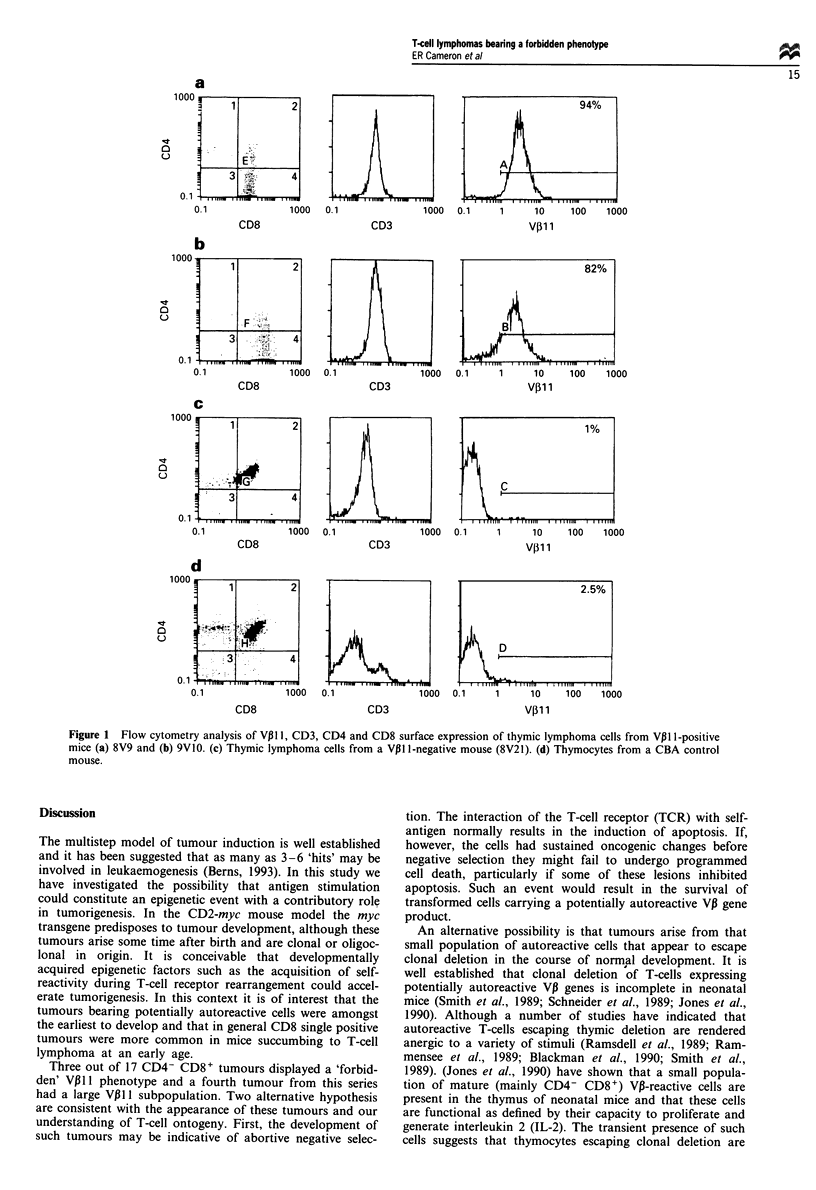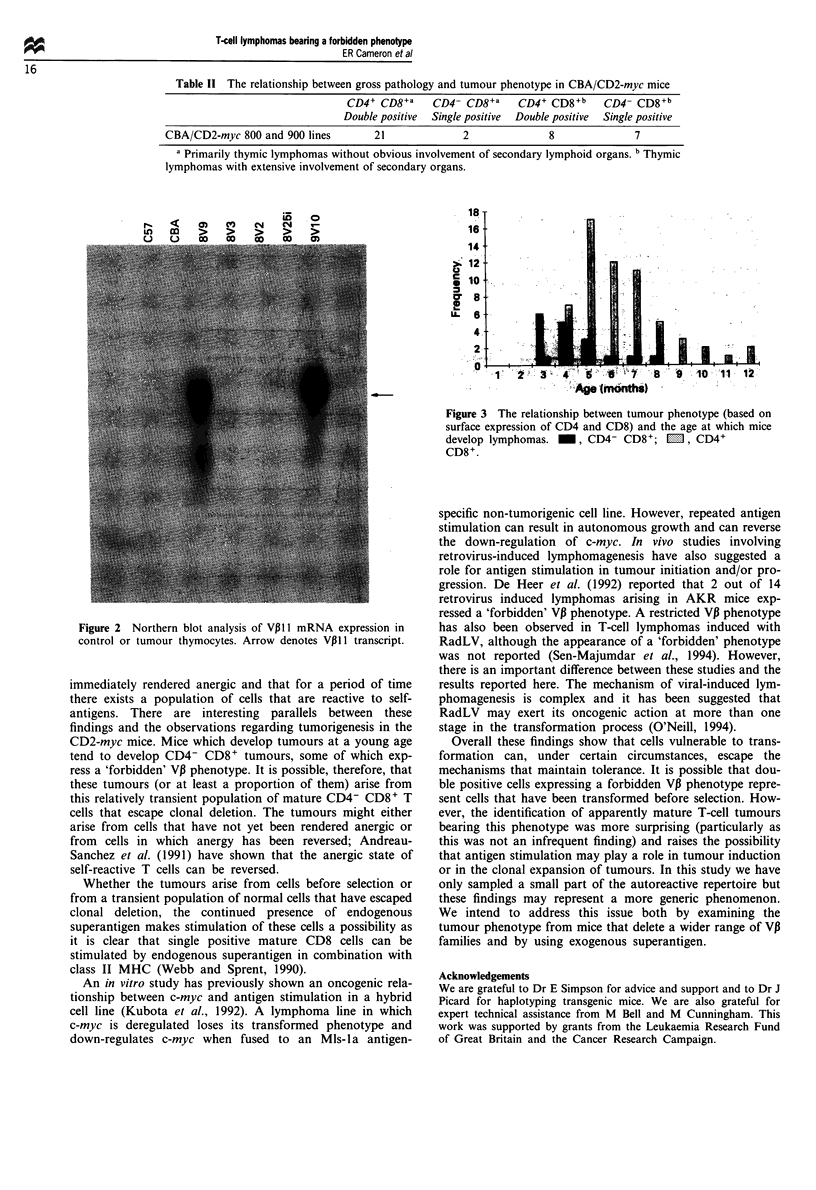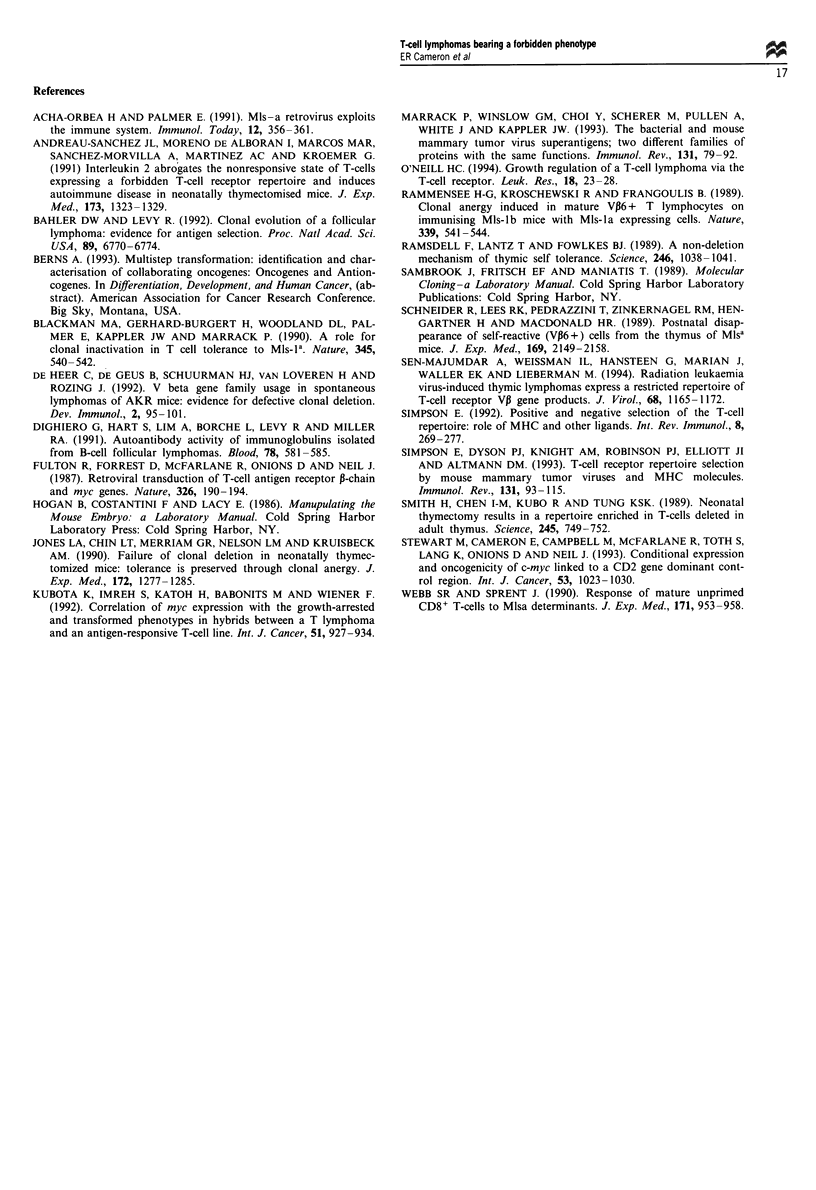# Apparent bypass of negative selection in CD8+ tumours in CD2-myc transgenic mice.

**DOI:** 10.1038/bjc.1996.3

**Published:** 1996-01

**Authors:** E. R. Cameron, M. Campbell, K. Blyth, S. A. Argyle, L. Keanie, J. C. Neil, D. E. Onions

**Affiliations:** Department of Veterinary Pathology, University of Glasgow Veterinary School, UK.

## Abstract

**Images:**


British Journal of Cancer (1996) 73, 13-17
© 1996 Stockton Press All rights reserved 0007-0920/96 $12.00

### Apparent bypass of negative selection in CD8$^+$ tumours in CD2-*myc* transgenic mice

ER Cameron[2], M Campbell[1], K Blyth[1,2], SA Argyle[2], L Keanie[1], JC Neil[1] and DE Onions[1]

*Departments of [1]Veterinary Pathology and [2]Clinical Studies, University of Glasgow Veterinary School, Bearsden Road, Glasgow G61 1QH, UK.*

**Summary** A role for antigen stimulation in lymphoid neoplasia has been postulated and is supported by indirect evidence that suggests that the interaction of antigen with both T cells and B cells may constitute an epigenetic event that can contribute to tumour induction or tumour progression. Using *myc*-bearing transgenic mice that develop mainly clonal T-cell lymphomas we have investigated the possibility that endogenous antigen-mediated clonal deletion might be overridden in tumorigenesis. CD2-*myc* transgenic mice were backcrossed on to a CBA/Ca background to ensure Mtv-mediated deletion of Vβ11-expressing T cells in the resultant offspring. Lymphomas arising from these mice were subsequently screened for Vβ11 expression. There was a clear correlation between the age at which mice developed neoplasia and the tumour phenotype. Mice with CD4$^-$ CD8$^+$ tumours succumbed to thymic lymphoma at a significantly younger age than mice developing CD4$^+$ CD8$^+$ tumours. A small number of tumours consisted of the 'forbidden' Vβ11 phenotype, showing that cells vulnerable to transformation could escape negative selection. The majority of the Vβ11-positive tumours were CD4$^-$ CD8$^+$ and were only observed in mice showing clinical evidence of tumour development at a relatively young age. The phenotype of these cells and the age at which tumours arose suggests that T cells escaping tolerance may be susceptible to transformation.

**Keywords:** T cell; lymphoma; Vβ phenotype; *myc*; transgene

There is now considerable evidence to suggest that the oncogenic transformation of healthy tissue involves a series of events, ultimately resulting in the appearance of the fully malignant phenotype. To study this process in the T-cell lineage we have produced two lines (800 and 900 series) of transgenic mice that harbour the human c-*myc* gene linked to the CD2 dominant control region. These CD2-*myc* transgenic mice display a moderate incidence of T-cell lymphoma development but it is obvious from the stochastic appearance and the clonal nature of these tumours that one or more secondary events are necessary for transformation to the fully neoplastic phenotype (Stewart *et al*., 1993). These mice are, therefore, a useful model with which to explore those oncogenic events that can cooperate with *myc* in T-cell leukaemogenesis. In this study, we investigated the possibility that antigenic stimulation of T cells through the T-cell receptor (TCR) might, under certain circumstances, complement the action of oncogenes such as *myc* in T-cell transformation.

The potential role of antigen stimulation in the transformation or clonal expansion of lymphoid tissue is not known but there is indirect evidence to implicate antigenic activation of lymphocytes in tumorigenesis. Follicular lymphomas are often committed to the production of autoantibodies and it has been suggested that B cells responsive to the continuous presence of self antigen are susceptible to transformation (Dighiero *et al*., 1991). Further, Bahler and Levy (1992) have reported that there is evidence for antigen selection in the development of follicular lymphomas. Analysis of a thymic lymphoma associated with a field case of feline leukaemia virus (FeLV) infection revealed the presence of a provirus which had incorporated a fully processed TCR gene, raising the possibility that antigen stimulation or inappropriate antigen recognition may have been important in the genesis of this tumour (Fulton *et al*., 1987). Studies involving murine radiation leukaemia virus (RadLV) have shown that free virus particles can modulate the growth of a RadLV-induced T-cell lymphoma cell line (O'Neill, 1994) and that the Vβ repertoire of RadLV-induced lymphomas is restricted, indicating non-random selection (Sen-Majumdar *et al*., 1994).

Correspondence: ER Cameron,
Received 6 April 1995; revised 17 July 1995; accepted 2 August 1995

The inbred mouse is a useful model with which to study endogenous antigen stimulation. Proviral mammary tumour virus (Mtv) sequences resident in the mouse genome comprise a polymorphic family of superantigens that react with different T-cell families defined by their Vβ gene usage and have, therefore, an important role in shaping the T-cell repertoire. Expression of the *sag* gene located in the 3' long terminal repeat (LTR) of endogenous mammary tumour proviral sequences results in the stimulation of mature T cells and in the clonal deletion of developing T cells bearing specific Vβ gene products (for reviews see Acha-Orbea & Palmer, 1991; Simpson, 1992; Marrack *et al*., 1993; Simpson *et al*., 1993).

We have exploited the presence of these endogenous superantigens to investigate the relationship between TCR Vβ restriction and lymphomagenesis using the tumour-prone CD2-*myc* transgenic mice. The appearance of tumours expressing a 'forbidden' Vβ phenotype would indicate that tumorigenesis bypasses or overrides negative selection. Here we present data to demonstrate that a small proportion of CD4$^-$ CD8$^+$ T-cell tumours can express a 'forbidden' Vβ11 phenotype.

### Materials and methods

### *Experimental animals*

The CD2-*myc* transgenic mice have been described previously (Stewart *et al*., 1993). The genetic background of these mice was a mixture of C57B16/J and CBA/Ca inbred lines. The transgenic mice were backcrossed to CBA/Ca mice and tumours arising in the offspring were analysed. This was carried out to ensure that the offspring all carried a functional I-Eα gene necessary for the deletion of autoreactive Vβ T-cell families. Mice developing lymphomas were culled as soon as clinical signs of malaise were apparent.

### *Hybridisation of DNA and RNA*

Preparation of high-molecular weight DNA from mouse tissues was carried out using standard protocols (Hogan *et al*., 1986). DNA was digested with the appropriate restriction endonucleases, separated by agarose gel electrophoresis, transferred to nylon filters and hybridised to the appropriate radiolabelled probes at high stringency using the protocols described by Sambrook *et al.* (1989). Random priming (Random Priming Kit, Amersham) and radiolabelling of these probes was also carried out using methods described by Sambrook *et al.* (1989). Transgene sequences were identified using a human c-*myc* exon 3 probe (1.38 kb, *Cla*I–*Eco*RI).

Cellular RNA was extracted from mouse tissues using the RNAzol B method (Biogenesis). Samples of 20 $\mu$g were separated by electrophoresis on 1% agarose gels containing 2.2 M formaldehyde, transferred on to nylon filters and hybridised using procedures described by Sambrook *et al.* (1989). V$\beta$11 expression was determined using a probe derived from a 0.4 Kb *Eco*RI/Sst fragment of the V$\beta$11 variable region (kindly supplied by Julian Dyson, MRC Clinical Research Centre, Harrow, UK).

*Flow cytometry*

Thymus tissue was mixed with phosphate-buffered saline and minced using sterile scissors and the dead cells removed by centrifugation through a Ficoll–hypaque gradient (2000*g* for 10 min). Cells were directly labelled by resuspending them at $2.5 \times 10^6$ ml with a 1:40 dilution of pretitrated antibody. Reaction mixtures (400 $\mu$l) were incubated at 4°C for 35 min before washing and analysis. Rat monoclonals FITC anti-CD8 (clone 53-6.7), RD anti-CD4 (clone YTS 191-1.2) and biotin anti-CD3 (clone YCD3-1) were obtained from Gibco. Rat anti-V$\beta$11 antibody (clone RR3-15) was obtained from Pharmingen.

### Results

*Small numbers of CD2-*myc*/CBA tumours expressed a forbidden V$\beta$11 phenotype*

CD2-*myc* transgenic mice harbour the human c-*myc* gene linked to the CD2 dominant control region and as a result these mice display a moderate incidence of spontaneous T-cell lymphoma. We sought to use these mice to investigate the influence of T-cell negative selection on tumour development. The presence of endogenous superantigens in mice results in the clonal deletion of specific V$\beta$ expressing families. We wished to establish the CD2-*myc* mice (originally derived from C57Bl/6 and CBA/Ca inbred strains) on a background that should result in the deletion of V$\beta$11 cells. CBA/Ca mice carry Mtv loci 8 and 9 together with a functional I-E gene (Simpson, 1992), by backcrossing onto CBA/Ca mice it was predicted that the offspring would delete V$\beta$11-expressing cells. To confirm that these $F_1$ mice could present endogenous superantigen a representative sample of the tumours was screened for the presence of an intact I-E$\alpha$ gene (J Picard, personal communcation). All CBA/CD2-*myc* backcrossed mice examined were found to have at least one allele with an intact I-E $\alpha$-chain gene. The potential role of autoreactive cells in lymphoma development was investigated by screening tumours for V$\beta$11 expression.

The phenotype of tumours arising from CBA backcrossed transgenic mice was investigated using flow cytometry. All tumours analysed were $CD3^+$ and the majority of tumours could be divided into two T-cell phenotypes. Nearly 70% of tumours were composed of cells that were predominantly $CD4^+$ $CD8^+$ and 20% were predominantly composed of cells of the $CD4^-$ $CD8^+$ phenotype (Table I). These results are similar to those previously observed in the parental CD2-*myc* mice (Stewart *et al.*, 1993). Backcrossing the mice did, however, result in an increased incidence of tumour development in both lines. In the 800 line and 900 lines the incidence rose from 19% (22/114) to 30% (102/340) and from 3% (2/66) to 34% (25/73) respectively.

Flow cytometry analysis of V$\beta$11 usage in tumour samples of CD2-*myc*/CBA mice showed that 3 of the 17 $CD4^-$ $CD8^+$ tumours analysed were largely composed of this 'forbidden' cell type and a fourth $CD4^-$ $CD8^+$ tumour contained a major subpopulation of V$\beta$11-positive cells. In contast V$\beta$11 cells were only found in 2 of the 61 $CD4^+$ $CD8^+$ tumours. To confirm that V$\beta$11 expression was present in these samples we analysed two of these tumours (8V9 and 9V10) for V$\beta$11 mRNA by Northern blot hybridisation using a V$\beta$11-specific probe, these results confirmed that both tumours expressed the V$\beta$11 transcript. The flow cytometry results and Northern blot results of these two tumours are shown in Figures 1 and 2 respectively. Therefore it appears that tumours representing T-cell families that would normally be deleted can be present in transgenic mice prone to thymic lymphoma development but their appearance is biased to the CD8 single positive subset.

*Tumour phenotype in CD2-*myc* mice was highly correlated with age*

Transgenic CBA backcrossed mice developed spontaneous thymic lymphomas from 2 months of age. The majority of mice that developed tumours did so between 3 and 9 months of age, with only a small proportion of mice greater than 9 months of age succumbing to lymphoma development. The CD4/CD8 phenotype of the T-cell tumours was closely correlated with the age at which mice develop thymic lymphoma. Mice developing thymic lymphoma at a comparatively young age were much more likely to be $CD4^-$ $CD8^+$ than mice developing lymphoma at an older age. The average age at which mice developed $CD4^-$ $CD8^+$ tumours was 115 days whereas the average age of mice developing $CD4^+$ $CD8^+$ tumours was 174 days. The difference between the two groups is highly significant (Mann–Whitney test, $P < 0.001$) and suggests that there is an age-related susceptibility to the development of $CD4^-$ $CD8^+$ tumours. The over-representation of $CD4^-$ $CD8^+$ tumours in young mice is shown in Figure 3.

The appearance of $CD4^-$ $CD8^+$ V$\beta$11 tumours was also correlated with age. The mice that developed V$\beta$11-expressing tumours of the $CD4^-$ $CD8^+$ phenotype were amongst the youngest mice to succumb to tumour development. Overall the average age at which mice develop thymic lymphoma is 161 days with a wide range from 65 to 333 days. The mice developing $CD4^-$ $CD8^+$ V$\beta$11-positive tumours all died between 65 and 94 days of age. The two mice that developed $CD4^+$ $CD8^+$ V$\beta$11-positive tumours died at 109 and 126 days.

In all of the tumours examined the thymus was greatly enlarged but gross pathological changes in the spleen and lymph nodes were much more variable. As a result the tumours could be broadly divided into two types: those that had only slight or no obvious splenic involvement and those that had gross involvement of the secondary lymphoid organs. There was a correlation between the phenotype of the tumours and the observed gross pathology. In those tumours in which gross involvement of secondary lymphoid organs was not obvious, 90% of tumours were $CD4^+$ $CD8^+$ and only 10% of tumours were $CD4^-$ $CD8^+$ whereas there was a far higher representation of $CD4^-$ $CD8^+$ tumours showing gross involvement of the secondary lymphoid organs. These results are shown in Table II.

**Table I** The phenotype of lymphoid tumours from the CD2-*myc* transgenic mice (800 and 900 lines) based on flow cytometry analysis of CD4 and CD8 expression.

| | *$CD4^+$ $CD8^+$ double positive* | *$CD4^-$ $CD8^+$ single positive* | *$CD4^+$ $CD8^-$ single positive* | *$CD4^-$ $CD8^-$ dougle negative* | *Mixed phenotype* |
|---|---|---|---|---|---|
| CBA/CD2-*myc* 800 and 900 lines | 61 | 18 | 1 | 1 | 8 |

**Figure 1** Flow cytometry analysis of Vβ11, CD3, CD4 and CD8 surface expression of thymic lymphoma cells from Vβ11-positive mice (**a**) 8V9 and (**b**) 9V10. (**c**) Thymic lymphoma cells from a Vβ11-negative mouse (8V21). (**d**) Thymocytes from a CBA control mouse.

### Discussion

The multistep model of tumour induction is well established and it has been suggested that as many as 3–6 'hits' may be involved in leukaemogenesis (Berns, 1993). In this study we have investigated the possibility that antigen stimulation could constitute an epigenetic event with a contributory role in tumorigenesis. In the CD2-*myc* mouse model the *myc* transgene predisposes to tumour development, although these tumours arise some time after birth and are clonal or oligoclonal in origin. It is conceivable that developmentally acquired epigenetic factors such as the acquisition of self-reactivity during T-cell receptor rearrangement could accelerate tumorigenesis. In this context it is of interest that the tumours bearing potentially autoreactive cells were amongst the earliest to develop and that in general CD8 single positive tumours were more common in mice succumbing to T-cell lymphoma at an early age.

Three out of 17 $CD4^-$ $CD8^+$ tumours displayed a 'forbidden' Vβ11 phenotype and a fourth tumour from this series had a large Vβ11 subpopulation. Two alternative hypothesis are consistent with the appearance of these tumours and our understanding of T-cell ontogeny. First, the development of such tumours may be indicative of abortive negative selection. The interaction of the T-cell receptor (TCR) with self-antigen normally results in the induction of apoptosis. If, however, the cells had sustained oncogenic changes before negative selection they might fail to undergo programmed cell death, particularly if some of these lesions inhibited apoptosis. Such an event would result in the survival of transformed cells carrying a potentially autoreactive Vβ gene product.

An alternative possibility is that tumours arise from that small population of autoreactive cells that appear to escape clonal deletion in the course of normal development. It is well established that clonal deletion of T-cells expressing potentially autoreactive Vβ genes is incomplete in neonatal mice (Smith *et al.*, 1989; Schneider *et al.*, 1989; Jones *et al.*, 1990). Although a number of studies have indicated that autoreactive T-cells escaping thymic deletion are rendered anergic to a variety of stimuli (Ramsdell *et al.*, 1989; Rammensee *et al.*, 1989; Blackman *et al.*, 1990; Smith *et al.*, 1989). (Jones *et al.*, 1990) have shown that a small population of mature (mainly $CD4^-$ $CD8^+$) Vβ-reactive cells are present in the thymus of neonatal mice and that these cells are functional as defined by their capacity to proliferate and generate interleukin 2 (IL-2). The transient presence of such cells suggests that thymocytes escaping clonal deletion are

**Table II** The relationship between gross pathology and tumour phenotype in CBA/CD2-*myc* mice

| | *CD4⁺ CD8⁺ᵃ* *Double positive* | *CD4⁻ CD8⁺ᵃ* *Single positive* | *CD4⁺* CD8⁺ᵇ *Double positive* | *CD4⁻* CD8⁺ᵇ *Single positive* |
|---|---|---|---|---|
| CBA/CD2-*myc* 800 and 900 lines | 21 | 2 | 8 | 7 |

[a] Primarily thymic lymphomas without obvious involvement of secondary lymphoid organs. [b] Thymic lymphomas with extensive involvement of secondary organs.

**Figure 2** Northern blot analysis of Vβ11 mRNA expression in control or tumour thymocytes. Arrow denotes Vβ11 transcript.

**Figure 3** The relationship between tumour phenotype (based on surface expression of CD4 and CD8) and the age at which mice develop lymphomas. ■, CD4⁻ CD8⁺; ▒, CD4⁺ CD8⁺.

immediately rendered anergic and that for a period of time there exists a population of cells that are reactive to self-antigens. There are interesting parallels between these findings and the observations regarding tumorigenesis in the CD2-*myc* mice. Mice which develop tumours at a young age tend to develop $CD4^-$ $CD8^+$ tumours, some of which express a 'forbidden' Vβ phenotype. It is possible, therefore, that these tumours (or at least a proportion of them) arise from this relatively transient population of mature $CD4^-$ $CD8^+$ T cells that escape clonal deletion. The tumours might either arise from cells that have not yet been rendered anergic or from cells in which anergy has been reversed; Andreau-Sanchez *et al.* (1991) have shown that the anergic state of self-reactive T cells can be reversed.

Whether the tumours arise from cells before selection or from a transient population of normal cells that have escaped clonal deletion, the continued presence of endogenous superantigen makes stimulation of these cells a possibility as it is clear that single positive mature CD8 cells can be stimulated by endogenous superantigen in combination with class II MHC (Webb and Sprent, 1990).

An *in vitro* study has previously shown an oncogenic relationship between c-*myc* and antigen stimulation in a hybrid cell line (Kubota *et al.*, 1992). A lymphoma line in which c-*myc* is deregulated loses its transformed phenotype and down-regulates c-*myc* when fused to an Mls-1a antigen-specific non-tumorigenic cell line. However, repeated antigen stimulation can result in autonomous growth and can reverse the down-regulation of c-*myc*. *In vivo* studies involving retrovirus-induced lymphomagenesis have also suggested a role for antigen stimulation in tumour initiation and/or progression. De Heer *et al.* (1992) reported that 2 out of 14 retrovirus induced lymphomas arising in AKR mice expressed a 'forbidden' Vβ phenotype. A restricted Vβ phenotype has also been observed in T-cell lymphomas induced with RadLV, although the appearance of a 'forbidden' phenotype was not reported (Sen-Majumdar *et al.*, 1994). However, there is an important difference between these studies and the results reported here. The mechanism of viral-induced lymphomagenesis is complex and it has been suggested that RadLV may exert its oncogenic action at more than one stage in the transformation process (O'Neill, 1994).

Overall these findings show that cells vulnerable to transformation can, under certain circumstances, escape the mechanisms that maintain tolerance. It is possible that double positive cells expressing a forbidden Vβ phenotype represent cells that have been transformed before selection. However, the identification of apparently mature T-cell tumours bearing this phenotype was more surprising (particularly as this was not an infrequent finding) and raises the possibility that antigen stimulation may play a role in tumour induction or in the clonal expansion of tumours. In this study we have only sampled a small part of the autoreactive repertoire but these findings may represent a more generic phenomenon. We intend to address this issue both by examining the tumour phenotype from mice that delete a wider range of Vβ families and by using exogenous superantigen.

**Acknowledgements**
We are grateful to Dr E Simpson for advice and support and to Dr J Picard for haplotyping transgenic mice. We are also grateful for expert technical assistance from M Bell and M Cunningham. This work was supported by grants from the Leukaemia Research Fund of Great Britain and the Cancer Research Campaign.

### References

ACHA-ORBEA H AND PALMER E. (1991). Mls-a retrovirus exploits the immune system. *Immunol. Today*, **12**, 356-361.

ANDREAU-SANCHEZ JL, MORENO DE ALBORAN I, MARCOS MAR, SANCHEZ-MORVILLA A, MARTINEZ AC AND KROEMER G. (1991) Interleukin 2 abrogates the nonresponsive state of T-cells expressing a forbidden T-cell receptor repertoire and induces autoimmune disease in neonatally thymectomised mice. *J. Exp. Med.*, **173**, 1323-1329.

BAHLER DW AND LEVY R. (1992). Clonal evolution of a follicular lymphoma: evidence for antigen selection. *Proc. Natl Acad. Sci. USA*, **89**, 6770-6774.

BERNS A. (1993). Multistep transformation: identification and characterisation of collaborating oncogenes: Oncogenes and Antioncogenes. In *Differentiation, Development, and Human Cancer*, (abstract). American Association for Cancer Research Conference. Big Sky, Montana, USA.

BLACKMAN MA, GERHARD-BURGERT H, WOODLAND DL, PALMER E, KAPPLER JW AND MARRACK P. (1990). A role for clonal inactivation in T cell tolerance to Mls-1[a]. *Nature*, **345**, 540-542.

DE HEER C, DE GEUS B, SCHUURMAN HJ, VAN LOVEREN H AND ROZING J. (1992). V beta gene family usage in spontaneous lymphomas of AKR mice: evidence for defective clonal deletion. *Dev. Immunol.*, **2**, 95-101.

DIGHIERO G, HART S, LIM A, BORCHE L, LEVY R AND MILLER RA. (1991). Autoantibody activity of immunoglobulins isolated from B-cell follicular lymphomas. *Blood*, **78**, 581-585.

FULTON R, FORREST D, MCFARLANE R, ONIONS D AND NEIL J. (1987). Retroviral transduction of T-cell antigen receptor β-chain and *myc* genes. *Nature*, **326**, 190-194.

HOGAN B, COSTANTINI F AND LACY E. (1986). *Manupulating the Mouse Embryo: a Laboratory Manual.* Cold Spring Harbor Laboratory Press: Cold Spring Harbor, NY.

JONES LA, CHIN LT, MERRIAM GR, NELSON LM AND KRUISBECK AM. (1990). Failure of clonal deletion in neonatally thymectomized mice: tolerance is preserved through clonal anergy. *J. Exp. Med.*, **172**, 1277-1285.

KUBOTA K, IMREH S, KATOH H, BABONITS M AND WIENER F. (1992). Correlation of *myc* expression with the growth-arrested and transformed phenotypes in hybrids between a T lymphoma and an antigen-responsive T-cell line. *Int. J. Cancer*, **51**, 927-934.

MARRACK P, WINSLOW GM, CHOI Y, SCHERER M, PULLEN A, WHITE J AND KAPPLER JW. (1993). The bacterial and mouse mammary tumor virus superantigens; two different families of proteins with the same functions. *Immunol. Rev.*, **131**, 79-92.

O'NEILL HC. (1994). Growth regulation of a T-cell lymphoma via the T-cell receptor. *Leuk. Res.*, **18**, 23-28.

RAMMENSEE H-G, KROSCHEWSKI R AND FRANGOULIS B. (1989). Clonal anergy induced in mature Vβ6+ T lymphocytes on immunising Mls-1b mice with Mls-1a expressing cells. *Nature*, **339**, 541-544.

RAMSDELL F, LANTZ T AND FOWLKES BJ. (1989). A non-deletion mechanism of thymic self tolerance. *Science*, **246**, 1038-1041.

SAMBROOK J, FRITSCH EF AND MANIATIS T. (1989). *Molecular Cloning-a Laboratory Manual.* Cold Spring Harbor Laboratory Publications: Cold Spring Harbor, NY.

SCHNEIDER R, LEES RK, PEDRAZZINI T, ZINKERNAGEL RM, HENGARTNER H AND MACDONALD HR. (1989). Postnatal disappearance of self-reactive (Vβ6+) cells from the thymus of Mls[a] mice. *J. Exp. Med.*, **169**, 2149-2158.

SEN-MAJUMDAR A, WEISSMAN IL, HANSTEEN G, MARIAN J, WALLER EK AND LIEBERMAN M. (1994). Radiation leukaemia virus-induced thymic lymphomas express a restricted repertoire of T-cell receptor Vβ gene products. *J. Virol.*, **68**, 1165-1172.

SIMPSON E. (1992). Positive and negative selection of the T-cell repertoire: role of MHC and other ligands. *Int. Rev. Immunol.*, **8**, 269-277.

SIMPSON E, DYSON PJ, KNIGHT AM, ROBINSON PJ, ELLIOTT JI AND ALTMANN DM. (1993). T-cell receptor repertoire selection by mouse mammary tumor viruses and MHC molecules. *Immunol. Rev.*, **131**, 93-115.

SMITH H, CHEN I-M, KUBO R AND TUNG KSK. (1989). Neonatal thymectomy results in a repertoire enriched in T-cells deleted in adult thymus. *Science*, **245**, 749-752.

STEWART M, CAMERON E, CAMPBELL M, MCFARLANE R, TOTH S, LANG K, ONIONS D AND NEIL J. (1993). Conditional expression and oncogenicity of c-*myc* linked to a CD2 gene dominant control region. *Int. J. Cancer*, **53**, 1023-1030.

WEBB SR AND SPRENT J. (1990). Response of mature unprimed $CD8^+$ T-cells to Mlsa determinants. *J. Exp. Med.*, **171**, 953-958.